# A Direct Comparison of Biplanar Videoradiography and Optical Motion Capture for Foot and Ankle Kinematics

**DOI:** 10.3389/fbioe.2019.00199

**Published:** 2019-08-23

**Authors:** Sarah E. Kessler, Michael J. Rainbow, Glen A. Lichtwark, Andrew G. Cresswell, Susan E. D'Andrea, Nicolai Konow, Luke A. Kelly

**Affiliations:** ^1^Centre of Sensorimotor Performance, School of Human Movement and Nutrition Sciences, The University of Queensland, Brisbane, QLD, Australia; ^2^Skeletal Observation Laboratory, Department of Mechanical and Materials Engineering, Queen's University, Kingston, ON, Canada; ^3^Department of Orthopaedics, Brown University, Providence, RI, United States; ^4^Department of Kinesiology, University of Rhode Island, Kingston, RI, United States; ^5^Providence VA Medical Center, Providence, RI, United States; ^6^Department of Biological Science, University of Massachusetts, Lowell, MA, United States

**Keywords:** foot, kinematics, ankle, biplanar videoradiography, motion analysis

## Abstract

Measuring motion of the human foot presents a unique challenge due to the large number of closely packed bones with congruent articulating surfaces. Optical motion capture (OMC) and multi-segment models can be used to infer foot motion, but might be affected by soft tissue artifact (STA). Biplanar videoradiography (BVR) is a relatively new tool that allows direct, non-invasive measurement of bone motion using high-speed, dynamic x-ray images to track individual bones. It is unknown whether OMC and BVR can be used interchangeably to analyse multi-segment foot motion. Therefore, the aim of this study was to determine the agreement in kinematic measures of dynamic activities. Nine healthy participants performed three walking and three running trials while BVR was recorded with synchronous OMC. Bone position and orientation was determined through manual scientific-rotoscoping. The OMC and BVR kinematics were co-registered to the same coordinate system, and BVR tracking was used to create virtual markers for comparison to OMC during dynamic trials. Root mean square (RMS) differences in marker positions and joint angles as well as a linear fit method (LFM) was used to compare the outputs of both methods. When comparing BVR and OMC, sagittal plane angles were in good agreement (ankle: R^2^ = 0.947, 0.939; Medial Longitudinal Arch (MLA) Angle: R^2^ = 0.713, 0.703, walking and running, respectively). When examining the ankle, there was a moderate agreement between the systems in the frontal plane (R^2^ = 0.322, 0.452, walking and running, respectively), with a weak to moderate correlation for the transverse plane (R^2^ = 0.178, 0.326, walking and running, respectively). However, root mean squared error (RMSE) showed angular errors ranging from 1.06 to 8.31° across the planes (frontal: 3.57°, 3.67°, transverse: 4.28°, 4.70°, sagittal: 2.45°, 2.67°, walking and running, respectively). Root mean square (RMS) differences between OMC and BVR marker trajectories were task dependent with the largest differences in the shank (6.0 ± 2.01 mm) for running, and metatarsals (3.97 ± 0.81 mm) for walking. Based on the results, we suggest BVR and OMC provide comparable solutions to foot motion in the sagittal plane, however, interpretations of out-of-plane movement should be made carefully.

## Introduction

The human foot is a complex anatomical structure, with 26 bones, and 33 articulating surfaces. Measurements of foot motion are complicated by the small size of many of the foot-bones as well as the complexity of their articular structures. Difficulties associated with accurately measuring foot motion have limited advancements in diagnosis and treatment of common foot pathologies. An accurate understanding of foot motion is crucial not only for the treatment of common pathologies, but for the advancement of research that aims to expand our current understanding of the foot.

Multi-segment foot models have become increasingly popular for describing foot motion during human walking or running (Leardini et al., 1999; Stebbins et al., 2006; Bruening et al., 2012; Kelly et al., 2014). These models divide the foot into multiple rigid segments to measure the motion of generalized foot regions, such as the calcaneus, mid-foot (cuneiforms, cuboid, and navicular), and metatarsals (Leardini et al., 1999; Oosterwaal et al., 2016). While dividing the foot into various segments allows for the estimation of movement of foot-bones that cannot be easily measured, this approach requires a number of assumptions that may lead to inaccuracies in kinematic, and kinetic measures (Nester et al., 2007; Zelik and Honert, 2018). The source of the inaccuracies is potentially 2-fold: the models do not account for individual bone-to-bone articulations (Leardini et al., 2007) and therefore may violate rigid-body assumptions, and the marker positions are prone to errors caused by soft-tissue artifact (STA) (Lundgren et al., 2008; Akbarshahi et al., 2010). Despite the limitations, multi-segment foot models are widely used due to the fact they are relatively straightforward to implement, with marker placements being reliable, and repeatable between operators (Simon et al., 2006; Stebbins et al., 2006; Caravaggi et al., 2011; Wright et al., 2011), and data collection and processing times being not overly arduous.

Biplanar videoradiography (BVR) has emerged as a viable tool to capture *in-vivo* foot-bone motion. This approach maps the position and orientation (pose) of three-dimensional (3D) bone models with two or more synchronized and calibrated x-ray image sequences of dynamic motions (Tashman and Anderst, 2003; Brainerd et al., 2010; Miranda et al., 2011, 2013). The mapping of 3D bones can be performed via two approaches: manual markerless tracking, or automatic tracking of implanted tantalum beads (Miranda et al., 2011, 2013). Tracking three-dimensional motion of tantalum beads using BVR is considered the gold standard for motion capture (Miranda et al., 2011), however given its invasive nature this approach is very difficult to evaluate across multiple individuals and hence is not considered here. Manual tracking approaches are slightly less accurate and can have translational errors ranging from 0.25 to 0.30 mm (Miranda et al., 2011) and rotational errors of 0.3 to 0.44° (Miranda et al., 2011). It is important to note, however, the methodologies applied in these studies are not susceptible to errors caused by bone occlusion, as tracking was performed on isolated bones (Miranda et al., 2011; Iaquinto et al., 2014). Therefore, it is possible the errors associated with manually-tracking dynamic, *in-vivo* BVR are higher than reported in current literature. While BVR provides an opportunity to study foot-bone motion during locomotion without the limitations of STA and rigid-body assumptions, it is not without its limitations, including: time intensive data processing (Miranda et al., 2011), a small field of view, the use of ionizing radiation, as well as the potential for operator-tracking errors (Anderst et al., 2009).

Both OMC and BVR may play an important role in assessing foot function; whether used in isolation or potentially used in tandem to overcome each other's limitations. However, before we can compare results from each system or use them in tandem to inform foot motion, we must first determine the convergent validity of the two systems. We must establish if kinematic measures of foot and ankle motion from OMC and BVR provide similar estimates of foot kinematics, or whether systematic differences might exist between the two technologies. Therefore, the aim of this study was to quantify the agreement between measures of ankle and medial longitudinal arch (MLA) motion using OMC and BVR. To compare systems, we created virtual motion capture markers using the manually-tracked (via scientific rotoscoping) bones of the foot from BVR. The virtual markers were then compared to their corresponding OMC markers, allowing for a direct comparison between the systems and an opportunity to assess the convergent validity of the systems. We hypothesized that the two systems, OMC and BVR, would produce similar ankle, and foot kinematics through the stance phase. However, small differences may emerge between individual marker trajectories from the two systems due to STA or manual BVR tracking errors.

## Methods

### Participants

Nine healthy participants originally volunteered to participate in the study (*n* = 9; males: 6, females: 3; height: 174 ± 8 cm; weight: 77 ± 13 kg). Participants were excluded if they had a history of lower limb injury or cardiovascular disease. Each participant read and completed an ethics approved consent form. The experimental protocols were approved in accordance with the ethical review guidelines at the University of Queensland (Ethics Approval Number: 2015000955) and the Providence Medical Center (Ethics Approval Number: NP52015022AP7815).

**Figure 1:**
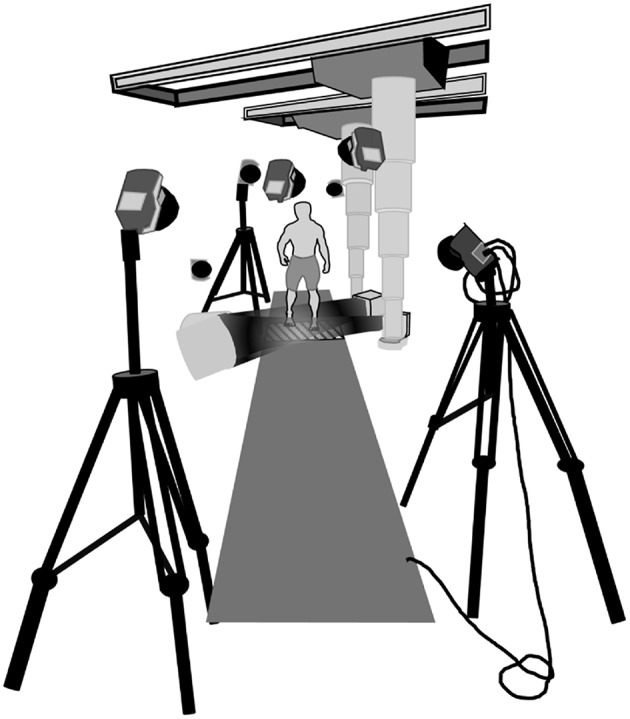
Schematic depiction of the laboratory set up. The two x-ray sources were set at a 130° angle ipsilateral to each other. The intensifiers were stationed as close to the translucent Dragon Plate (AllRed and Associates, Elbridge, USA) as possible while maintaining visibility of the foot. The entire platform was evenly raised 0.60 m from the ground. Eight OMC cameras were stationed around the collection space to optimize viewing of the foot markers.

Participants were provided as much time as they required to familiarize themselves with walking and running along a raised walkway (height: 0.6 m) in the laboratory (Figure 1). They were instructed to perform walking and running trials at a self-selected pace, with the starting position on the walkway designated to ensure that each participant's right heel landed in the middle of the x-ray collection space without altering their natural gait. A maximum of three walking trials and three running trials were collected per participant. If a participant's foot did not land in the x-ray capture space during a walking or running trial, data from that trial was discarded from the analysis. Data analysis was performed on seven of the nine healthy participants (five males, two female), with two participant's data not analyzed due to issues arising with data reconstruction. In total, 17 walking and 14 running trials were analyzed.

### Data Collection

**Figure 2:**
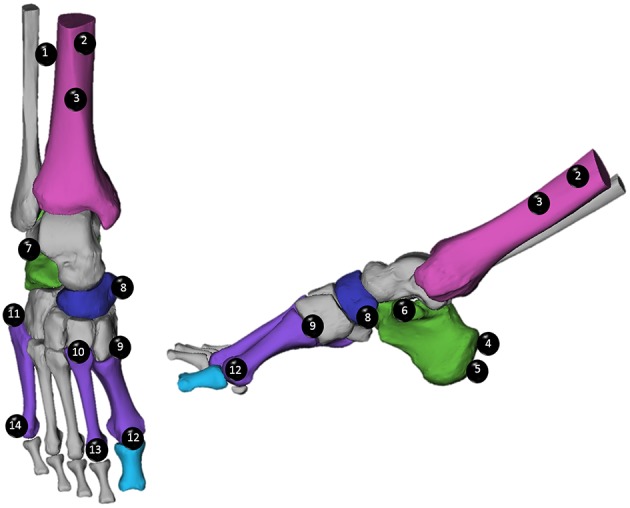
Optical motion capture (OMC) marker placement was performed in accordance with the Rizzoli model (Leardini et al., 2007). Markers are listed in order from proximal to distal, and numbered, to assist with identification. Tibial Markers (Magenta): (1) Lateral Shank (LtS), (2) Medial Shank (MdS), (3) Distal Shank (Shk). Calcaneal Markers (Green): (4) Superior Calcaneal Ridge, (5) Inferior Calcaneal Ridge (ICR), (6) Sustentaculum Tali (ST), (7) Peroneal Tubercle. Navicular Marker (Blue): (8) Navicular (TN). Metatarsal Markers (Purple): (9) First Metatarsal Base (FMB), (10) Second Metatarsal Base (SMB), (11) Fifth Metatarsal Base (VMB), (12) First Metatarsal Head (FMH), (13) Second Metatarsal Head (SMH), (14) Fifth Metatarsal Head (VMH).

14 retro-reflective markers were placed on anatomical landmarks on the participant's right foot and shank, in accordance with a previously described multi-segment foot model (Figure 2; Leardini et al., 2007). 3-Dimensional (3D) marker positions were captured using an OMC system (Qualysis Track Manager, Qualysis AB, Gothenburg, SWE) sampled at 250 Hz.

A high-speed, BVR method was used to simultaneously record x-ray images of the foot across a capture volume that was in the middle of the OMC space. The BVR system, X-ray Reconstruction of Moving Morphology (XROMM), has been previously documented by Brainerd et al. (2010) and Knorlein et al. (2016) for validation of beaded tracking. This BVR method consisted of two x-ray transmitters paired with two x-ray receivers that were coupled to high-speed cameras. The transmitters emitted x-rays using an exposure of 75 kVp and 100 mA. Lateral-oblique views were obtained by setting the imaging planes at 130° relative to each other about a global vertical axis. A carbon fiber plate (Dragon Plate, AllRed and Associates, Elbridge, USA) was placed in the middle of the capture volume, which allowed for enhanced bone visibility in the x-ray images. Participants landed with their right foot on the plate during each trial. The high-speed camera captured the x-ray images at 250 Hz. BVR data collection was manually triggered just prior to the participant's right foot entering the data collection space and was terminated when the participant's foot was no longer in the field of view. A synchronization signal from the x-ray system was used to trigger the start of the OMC system, which was recorded with a 0.5 s pre-trigger, and a trial duration of 3.0 s.

#### Computed Tomography (CT)

A computed tomography (CT) scan of each participant's right foot was captured with the participant lying prone with the ankle in a plantarflexed orientation (average resolution: 0.419 mm × 0.419 mm × 0.625 mm, LightSpeed 16, GE Medical Systems, USA). This orientation was chosen to improve the in-plane resolution which aids in the segmentation process. Foot position was maintained during the scan via a custom-made foam support. Two participants received CT scans at a different imaging location (for convenience) and therefore had a different CT protocol (resolution: 0.488 mm × 0.488 mm × 0.312 mm, Optima CT 660, LightSpeed CT, GE Medical Systems, USA). Each bone of the foot was segmented using Mimics17 (Materialize, Leuven, Belgium). The segmentation provided a tessellated 3D surface mesh, as well as, a 3D partial volume for each bone of interest.

### Data Processing

#### Motion Capture Data

The OMC markers were digitized using proprietary software (Qualysis Track Manager, Qualysis AB, Gothenburg, SWE), and exported to Matlab (R2016b, Mathworks, Nattick, USA). The data was filtered using a dual low-pass second order Butterworth filter with a cut-off frequency of 10 Hz. A custom-written software program was applied to determine heel-strike and toe-off by differentiating the coordinates of the inferior calcaneal ridge marker (ICR) to provide vertical marker velocity. In cases where the ICR marker was occluded, the superior calcaneal ridge marker (SCR) was used. The minimum velocity signified heel-strike and the maximum velocity signified toe-off (Ghoussayni et al., 2004).

#### Biplanar Videoradiography Data

Images obtained with BVR are subject to distortion when x-ray beams are transformed into visible images (Tersi and Stagni, 2014). To correct for the distortion, an x-ray image was collected with an “un-distortion” grid (Brainerd et al., 2010). The grid consisted of a perforated piece of sheet metal with known size and spacing of each hole (Brainerd et al., 2010). A software program, XMALab, previously described by Knorlein et al. (2016), used a distortion-correcting algorithm to correct for any changes to spacing or size of the holes in the perforated grid (XMALab, Brown University, RI, USA).

The calibration of the BVR system has been previously documented (Knorlein et al., 2016). Briefly, a custom calibration cube was used to calibrate the 3D volume of the BVR system. The cube consisted of four sheets of plexiglass separated by nylon tubes. Each layer of plexiglass had 16 holes equally spaced apart with 3 mm radio-opaque beads placed in each hole. To orient the cube, there were four metal objects placed inside the levels of plexiglass with their exact location known. Images were taken of the cube. XMALab determined the locations of the radio-opaque beads within the field of view in the cube images and computed the camera calibration matrices (Brainerd et al., 2010; Knorlein et al., 2016).

**Figure 3:**
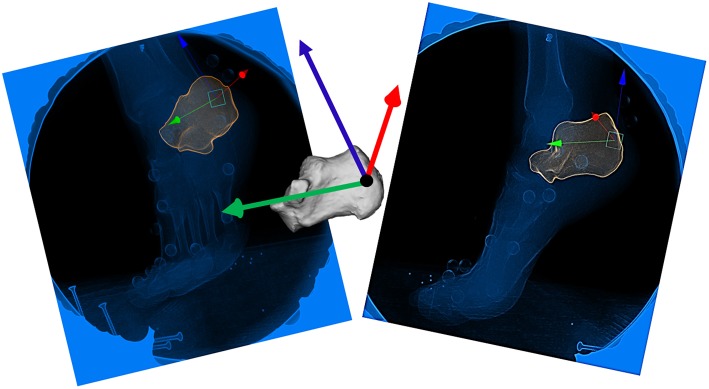
Scientific Rotoscoping: Depiction of the manual alignment of the calcaneus just prior to toe off. Each bone is aligned with the bony landmarks visible on the x-ray images in both cameras (camera 1, left and camera 2, right). Once the 3D bone is simultaneously aligned within both camera 1 and camera 2, it is considered “tracked” for that frame.

The calibration, undistorted x-ray images, and partial volumes for the bones of interest were imported into Autoscoper, a previously-described software for 3D markerless bone tracking (Miranda et al., 2011). The position and orientation of each bone was determined via scientific rotoscoping (Gatesy et al., 2010). Scientific rotoscoping is a process by which the 3D partial volume of the bone of interest is virtually placed in the BVR volume and a digitally reconstructed radiograph (DRR) is computed by simulating x-rays from the calibrated sources. The DRR is projected onto the x-ray image sequences and the partial volume is rotated and translated until the two DRRs (one for each x-ray image) match the captured x-ray images (Figure 3). This process yields six-degree-of-freedom transformation matrices that move each bone from the CT-based global coordinate system to the BVR-based global coordinate system. After the initial manual guess, the position and orientation of the bone was optimized by minimizing the normalized cross-correlation between the DRRs and the x-ray images across both views using a downhill simplex algorithm. This process was repeated every 10 frames and the remaining frames were interpolated using a quaternion spline. If the DRR for an optimized frame was misaligned, the bone was manually retracked to correct the placement, and the spline was recomputed. All tracking was performed on one bone at a time for as much of the stance phase that the bone was visible in both cameras. The transformation matrices were then converted to quaternions and filtered using a dual low-pass second order Butterworth filter with a cut off frequency of 10 Hz in Matlab (R2016b, Mathworks, Natick, USA). Using the same gait events, determined by the OMC heel-strike detection algorithm, the BVR data was isolated from heel-strike to toe-off, and interpolated to 101 points. If the bone could not be tracked at any point from heel-strike to toe-off due to lack of visibility, the non-tracked frames were not used in subsequent analyses.

#### Synchronization of Technologies

A synchronization test was used to determine discrepancies in sampling frequency between OMC and BVR. The test required dropping a retro-reflective marker into the capture volume and simultaneously recording OMC and BVR. This task was repeated five times. The marker was digitized in Qualysis, as well as, XMALab. The two technologies were aligned when the vertical coordinate (Z-coordinate) of the marker was at its minimum in both data sets (indicating impact). As a result, we determined there to be a slight time offset and, as such, OMC was down sampled to 99% of the original sample frequency to match the BVR data.

#### Co-registration and Creation of Virtual Markers

The OMC and BVR datasets were transformed into the same coordinate system, using a co-registration procedure (Miranda et al., 2013). A custom-built calibration object with 11 steel beads placed at the center of spherical retro-reflective markers was simultaneously captured by both the OMC and BVR systems. A minimum of three non-collinear markers were used to find the least squares fit to create a rigid-body transformation matrix (Soderkvist and Wedin, 1993). The resultant transformation matrix was used to transform the BVR data from the x-ray coordinate system into the OMC coordinate system.

The scientifically rotoscoped BVR data was then used to create virtual markers on each bone, aligned to the location of the skin-mounted markers in a static standing trial. The virtual markers were expressed in the reference frame of their parent bone, such that the 3D position could be calculated based on the movement of the scientifically rotoscoped bone. The 3D position of the BVR markers were then compared to the OMC markers in the same coordinate system.

### Data Analysis

#### Ankle Angle and Medial Longitudinal Arch Angle Calculations

Optical (OMC) and virtual (BVR) marker data was used to measure ankle angle (motion of the calcaneus relative to the shank) and MLA angle (sagittal plane: motion of the first metatarsal head relative to the calcaneus) to directly compare the angles measured by the two systems. All segments were created based on a previously described Rizzoli multi-segment foot model (Leardini et al., 2007). Rotations about the ankle were examined across all three planes and defined in accordance with the recommendations of the International Society of Biomechanics: sagittal plane (z-axis rotations), frontal plane (x-axis rotations), and transverse plane (y-axis rotations) (Wu and Cavanagh, 1995; Sinclair et al., 2012, 2013).

MLA angle was calculated using the vectors formed by the inferior calcaneal ridge to sustentaculum tali and the sustentaculum tali to first metatarsal head, (∢$\vec{\text{ICRST}};\;\vec{\text{STFMH}}$). The angle between the vectors was projected onto the plane orthogonal to the plane of the foot. The plane of the foot was established using the sustentaculum tali to the first metatarsal head markers to define the “x” axis, and the sustentaculum tali to the peroneal tubercle markers were deemed the “y” axis.

Ankle angle and MLA angle were only averaged across periods where bones were tracked for all trials. This resulted in ankle angle and MLA angle being processed from 11% of stance through to 75% of stance for all trials.

#### Marker Trajectory Differences

The root mean square (RMS) difference in 3D position between virtual markers created from BVR measurements and the OMC marker positions was calculated during the stance phase of each walking and running trial.

$$RMS\;=\;\sqrt{mean\left({\left(OMC\;-\;BVR\right)}^{2}\right)\;}$$

The RMS equation was then used for each individual plane to provide directional differences of marker trajectories in each axis in the global coordinate system (X—anterior-posterior, Y—medial-lateral, Z—superior-inferior).

As the capture volume for BVR was only slightly larger than the foot, the entire stance phase (e.g., foot contact to toe-off) was sometimes not captured, with a small period of data missing either at the beginning or end of stance. To mitigate this issue, walking and running trials were only analyzed from the first time point where tracking was present across all trials, and analysis concluded at the final time point where tracking was present for all trials, for each individual participant. This process provided an average RMS difference across stance per marker for walking and running for each participant.

#### Statistics

To quantify the agreement between ankle and MLA angles output by BVR and OMC, we used two statistical methods: a linear fit model (LFM) and a root mean squared error (RMSE). (A visualization of the LFM of one participant's data is represented in Supplementary Figures 3, 4). The use of LFM to compare kinematics has been well-documented by Iosa and Cappozzo as a reliable, sensitive and specific means of comparing gait waveforms (Iosa et al., 2014). The LFM assesses waveform similarities via a strength of linear fit (R^2^), offsets or shifts (a0), and variation of one wave relative to another (a1) (Iosa et al., 2014). If the two systems were to produce identical waveforms, we would anticipate the following values: R^2^ = 1, a0 = 0, and a1 = 1, RMSE = 0.0°. R^2^ relationships range from 0.0 to 1.0 (no relationship = 0.0 to 0.3, weak = 0.3 to 0.5, moderate = 0.5 to 0.7, and strong = 0.7 to 1.0) (Zikmund, 2000; Mukaka, 2012). Kinematic waveforms were compared from 11% of stance through to 75% of stance for all trials.

A Shapiro-Wilk Test of Normality was performed to determine normality of the distribution of the RMS differences in marker trajectories. RMS trajectory differences (walking and running) and marker trajectory directional differences (walking and running) were not normally distributed (P ≤ 0.05). As such, a non-parametric *t*-test was used to compare RMS differences in marker trajectories in walking and running. A non-parametric Kruskal-Wallis test was used to determine the significance of directional differences in marker trajectories in walking and running.

## Results

### Ankle Angle

**Figure 4:**
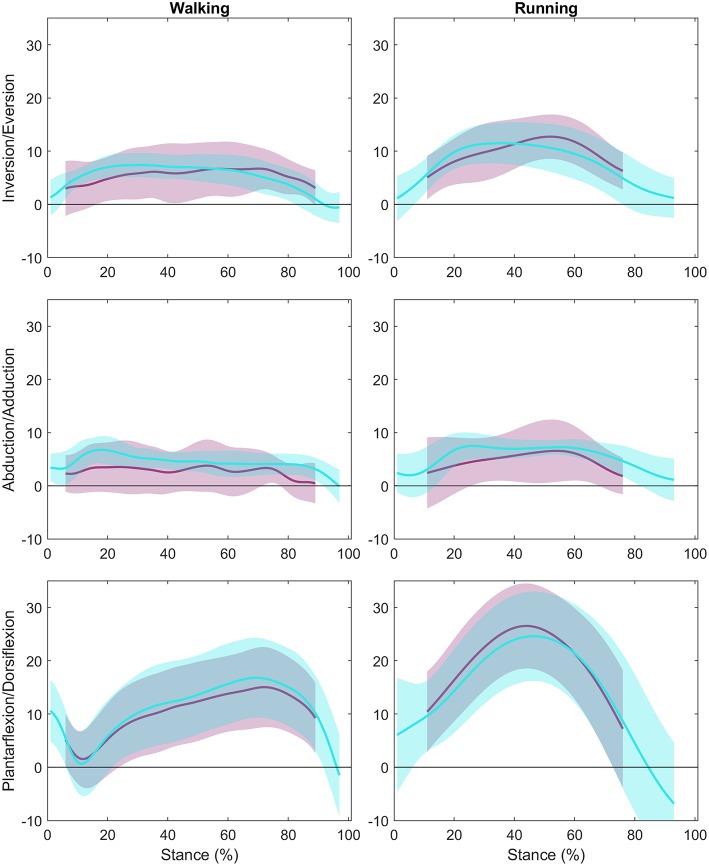
Ankle Angle Group Means: The group mean ankle angle across stance with the first column showing walking data, and the second column representing running data. The y-axes represent the three rotations (Inversion/Eversion, Abduction/Adduction, and Plantarflexion/Dorsiflexion), and the x-axes represent percent of stance. The optical motion capture (OMC) ankle angle ± 1 SD is represented in teal while the biplanar videoradiography (BVR) ankle angle ± 1 SD is displayed in light purple.

Group mean ankle angle during the stance phase for walking and running is presented for all three anatomical planes in Figure 4 (individual participant data is shown in Supplemental Figure 1). The LFM of ankle angles produced using OMC and BVR varied between anatomical planes and ranged from weak (0.178) to strong (0.947) (Table 1). The weakest LFM fit was found in the transverse plane for both walking and running (R^2^ = 0.178, range: 0.02–0.50, walking; R^2^ = 0.326; range: 0.04–0.58, running). Sagittal plane angles demonstrated the strongest R^2^ fit within the LFM (R^2^ = 0.947, range: 0.9–0.98, walking; R^2^ = 0.939, range: 0.87–0.99, running). When examining potential offset or phasic shifts of the waveforms produced by the two systems, no clear pattern emerges, with seemingly random offset values ranging from −4.7 to 9.0°.

**Table 1:** Waveform analysis of ankle angle and Medial Longitudinal Arch (MLA) angle produced by optical motion capture (OMC) and biplanar videoradiography (BVR) using a linear fit model (LFM) and root mean squared error (RMSE).

	***LFM*** **R^2^**	***LFM*** **a_0_ (^°^)**	***LFM*** **a_1_ (slope)**	***RMSE*** **(^°^)**
**WALKING**				
**Ankle**				
Inversion/Eversion:	**0.322** (0.16, 0.47)	**5.491**° (3.28°, 8.53°)	**0.079** (−0.66, 0.38)	**3.574**° (1.51, 5.16)
Abduction/Adduction:	**0.178** (0.02, 0.50)	**4.234**° (−0.23°, 6.60°)	**0.212** (−0.01, 0.81)	**4.281**° (2.21, 6.29)
Plantarflexion/Dorsiflexion:	**0.947** (0.9, 0.98)	–**0.175**° (−3.89°, 3.58°)	**1.146** (1.03, 1.39)	**2.446**° (1.05, 4.22)
**MLA**				
Angle:	**0.719** (0.58, 0.80)	**1.60**° (0.11°, 2.52°)	**0.538** (0.37,0.80)	**2.463**° (1.12°, 3.94°)
**RUNNING**				
**Ankle**				
Inversion/Eversion:	**0.452** (0.02, 0.90)	**4.539**° (−2.30°, 8.96°)	**0.403** (−0.28, 0.75)	**3.668**° (1.46°, 5.49°)
Abduction/Adduction:	**0.326** (0.04, 0.58)	**5.500**° (0.90°, 8.77°)	**0.266** (−0.15, 0.57)	**4.704**° (2.57°, 8.31°)
Plantarflexion/Dorsiflexion:	**0.939** (0.87, 0.99)	**0.825**° (−4.67°, 4.60°)	**0.915** (0.82, 1.07)	**2.670**° (1.06°, 4.17°)
**MLA**				
Angle:	**0.672** (0.54, 0.82)	**1.894**° (−4.09°, 4.45°)	**0.844** (0.68, 1.26)	**2.927** (2.27°, 3.92°)

*The results compare waveforms across stance for 17 walking trials and 14 running trials. R^2^ represents the correlation between the waveforms, a_0_ represents an offset or shift between the data, a_1_ represents the variation of the datasets. RMSE represents the difference in 3D space between the angles. Bold values represent the group mean, while parenthetical values represent the range*.

The RMSE, a measure of 3D differences between ankle angles, produced the smallest difference in the sagittal plane for walking and running (2.45 and 2.67°, walking and running, respectively). The largest RMSE differences were in the transverse plane for both walking and running (4.28 and 4.70°, respectively).

### MLA Angle

**Figure 5:**
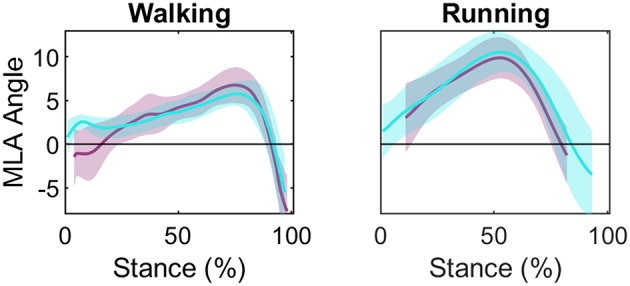
Group mean (± 1 SD) medial longitudinal arch (MLA) angle during stance phase for walking and running: optical motion capture (OMC) MLA angle (teal) and biplanar videoradiography (BVR) MLA angle (light purple). The y-axis represents MLA normalized to MLA at static. The x-axis represents percent of stance. General patterns of motion appear similar despite an offset in initial angle.

Group mean MLA angle during stance phase is presented in Figure 5 (individual participant data is shown in Supplemental Figure 2). The LFM for the MLA angle produced by OMC and BVR displayed moderate-to-strong similarity (R^2^ = 0.672 and 0.719, for walking and running, respectively) (Table 1). The offset for MLA angle during running trials displayed considerable variability (range: −4.09 to 4.45°). However, for walking trials the MLA angle offset was less variable (range: 0.11 to 2.52°).

### Marker Trajectory Differences

The group mean RMS differences in trajectories between OMC and BVR are shown for each marker in Table 2. The RMS differences between OMC and BVR markers were significantly larger when running compared to walking (*P* < 0.001). The shank and first metatarsal possessed the greatest RMS differences (shank: 3.35 mm and 6.00 mm, first metatarsal: 3.97 mm and 4.55 mm walking and running, respectively). The navicular (3.04 and 3.79 mm, walking and running, respectively) and calcaneus (2.99 and 3.93 mm, walking and running, respectively) were found to have the smallest RMS difference between modalities.

**Table 2:** Root Mean Squared (RMS) differences in marker trajectories (± 1SD) produced by optical motion capture (OMC) and biplanar videoradiography (BVR) with differences expressed in millimeters (mm).

**Marker**	**Walking**				**Running**			
	**RMS ± 1SD (mm)**	**Directional differences (mm)**			**RMS ± 1SD (mm)**	**Directional differences (mm)**		
		**X**	**Y**	**Z**		**X**	**Y**	**Z**
SCR	3.60 ± 1.47	2.94	3.18	2.59	4.21 ± 1.34	4.22	3.11	3.4
ICR	2.86 ± 1.06	2.14	2.65	1.9	3.92 ± 0.91	3.39	2.62	3.93
ST	2.74 ± 0.73	3.2	1.93	2.06	3.79 ± 0.78	3.73	2.46	3.51
PT	2.70 ± 1.11	3.03	1.37	2.33	3.96 ± 1.44	3.94	1.88	4.12
TN	3.04 ± 0.69	3.32	1.91	2.76	3.79 ± 0.82	3.51	1.91	4.32
FMB	4.02 ± 0.69	2.92	3.87	3.78	4.78 ± 1.12	3.93	4.14	4.5
FMH	3.93 ± 0.98	3.53	3.42	3.37	4.33 ± 1.35	3.54	2.96	4.77
LtS	3.72 ± 1.82	3.58	3.95	1.83	6.21 ± 1.97	8.12	3.71	3.71
MdS	3.72 ± 1.60	3.39	4.1	1.59	6.54 ± 2.26	8.01	3.91	4.46
ShK	2.62 ± 1.31	2.64	2.58	1.29	5.27 ± 1.86	6.37	2.44	4.26

*Marker Trajectory acronyms are as follows: Calcaneal Markers - superior calcaneal ridge (SCR), inferior calcaneal ridge (ICR), sustentaculum tali (ST), peroneal tubercle (PT). Midfoot Marker: Navicular (TN). Metatarsal Markers: first metatarsal base (FMB), first metatarsal head (FMH). Shank Markers: lateral shank (LTS), medial shank (MdS), distal shank (ShK). For marker placement reference, please refer to Figure 2*.

The group mean marker trajectory directional differences are shown in Table 2. When separating the RMS differences into directional components, there was no significant difference between the XYZ components during walking (*P* = 0.73). The RMS difference was significantly different between the XYZ components when running (*P* ≤ 0.05), with the largest differences observed in the medial-lateral direction and the smallest differences in the anterior-posterior direction.

## Discussion

We created an approach to directly, and non-invasively, compare foot motion measurements based on trajectories of skin-mounted markers (OMC) and trajectories of the same markers, virtually attached to the underlying bones (BVR). As hypothesized, we observed differences between the skin-mounted and virtual marker trajectories for all markers, with the magnitude of difference varying with each bone. We observed relatively small absolute differences in joint angles produced by the two systems across all planes of motion. The angles produced in the primary plane of motion (sagittal plane) for the ankle and MLA displayed strong agreement for both walking and running, however where there was little overall motion (e.g., ankle transverse plane) the agreement in waveforms was weaker. Our data suggests that the two motion capture technologies produce similar kinematic outputs during walking and running in the sagittal plane, while other planes show larger relative differences.

### Ankle and Medial Longitudinal Arch Motion

The primary purpose of this study was to determine if measures of ankle and MLA motion were in agreement when quantified using two different motion capture technologies. Our data suggests that the plane in which angular rotations are measured has a large influence on the similarity of angular outputs produced from the OMC and BVR systems. Sagittal plane angles were found to have a strong level of agreement, in the absence of specific offsets in amplitude or phase shifts. Conversely, angles produced at the ankle in the frontal plane and transverse plane displayed weak to moderate levels of shape similarity and a wide range of a0/a1 values, suggesting that these waveforms vary in both shape, magnitude and offset. Furthermore, given the variance of angle offset values, and lack of trend, it is likely the difference between the systems is movement dependent and participant specific.

It is important to note that the absolute differences in angles produced by the two systems was rather consistent across all planes (mean RMSE of 2.45–4.70°). This suggests that the small range of motion of the ankle in the frontal and transverse planes is a contributing factor to the reduced agreement in waveforms in these planes. For example, small fluctuations in angles arising from STA or bone alignment error, may have a larger influence on the overall waveform for the transverse plane, but not the sagittal, due to the much greater range of motion in the sagittal plane. Therefore, the overall foot posture is in good agreement between OMC and BVR.

The angle produced at the MLA was also generally similar in magnitude between the two technologies. The R^2^ for walking and running suggests a strong correlation between MLA angles assessed using OMC and BVR. Interestingly, for walking, the Y-intercept was positive for 90% of the trials. This would be indicative of a smaller MLA angle at 11% of stance when measured with BVR. It is possible that the smaller MLA angle measured by BVR may be a result of the deformation of the heel pad, which has been shown to deform between 10 and 12 mm (De Clercq et al., 1994; Wearing et al., 2009). This compression of the heel pad has been shown to occur rapidly, in the first 50 ms following heel strike. It is likely given the magnitude and the rate of change, this compression of the heel pad is otherwise indistinguishable using motion capture (De Clercq et al., 1994) and resulting in an overestimation of MLA immediately following heel strike.

### Marker Trajectory Differences

RMS differences between the two measurement methods were task dependent, with differences being slightly higher for running compared to walking (mean RMS: 3.29 ± 0.55, 4.68 ± 1.01 mm, walking and running, respectively). In both walking and running, the larger errors were seen in the shank markers. It is likely the larger magnitude of trajectory differences in the shank is attributable to greater soft tissue mass and the associated movement of the skin mounted marker relative to the leg. On average RMS differences reported here are slightly lower than current literature values obtained from bone pin studies, that report STA errors ranging from 10 to 27 mm (Benoit et al., 2006; Akbarshahi et al., 2010). Potentially, the current literature values have resulted in higher STA errors due to the use of bone-pins, which have been suggested to sustain vibrations or bending when a participant walks or runs (Ramsey et al., 2003). Thus, the bone-pins themselves could be responsible for registering higher STA error.

The magnitude of planar differences for marker trajectories were also task dependent. Walking produced the largest errors in the anteroposterior direction whereas the smallest were found for the superoinferior direction. Conversely, in running, the greatest errors were observed in the anteroposterior plane and the smallest errors were in the mediolateral plane. The magnitude observed in directional differences for walking is consistent with the single-plane videoradiography data computed by Akbarshahi et al. (2010). While the general pattern of directional differences in our data are similar to those of Akbarshahi et al. (2010), the magnitude of difference in our data is noticeably smaller. These differences are most likely due to the Akbarshahi study using a single plane approach, which the authors cited as introducing rotoscoping errors up to 3 mm (Akbarshahi et al., 2010). While the planar differences for all marker trajectories occurred in all directions, it is important to note the magnitude of these differences are minimal (average: 2.35–4.87 mm).

### Implications for Future Research

Comparing *in-vivo* bone-motion to bone-motion estimated from OMC presents a unique insight into foot biomechanics. Our data demonstrates that OMC and BVR produce similar results for standard gait analysis, particularly in the sagittal plane during the period of stance analyzed (11–75%). These results suggest that BVR and OMC can equally be used to inform sagittal plane motion of current foot models. BVR provides advantages in being able to directly quantify motion of joints that have bones that are almost impossible to track directly with skin-mounted markers (e.g., subtalar and talonavicular joint). In contrast, OMC is sufficient for basic foot and ankle motion estimates, such as those explored here. Optical motion capture can be used to measure kinematics across multiple full strides, as opposed to partial strides often only available with BVR. Synchronous use of the two systems to explore specific joint function (BVR) in the context of general foot motion (OMC) would keep processing times shorter while enhancing understanding of individual joint function. Equally, OMC could be used to provide valuable initial guesses of bone position and orientation in the BVR, such that optimisation approaches could be used to inform the rotoscoping procedure.

Despite the sagittal plane agreement, the frontal and transverse plane dissimilarities are of some concern. The source of the divergence between the systems is unclear, and this research demonstrates that more work needs to be done to determine why the kinematics vary depending on the system recording them. It is possible the differences stem from BVR being more sensitive to bone movement, and therefore, a more accurate measure of kinematics not susceptible to STA. However, it is also likely that some of the differences are the result of scientific rotoscoping errors in BVR where poor bone alignment could create movement artifacts. We note that the image intensifiers were oriented primarily in the sagittal plane, which may affect the ability to resolve the orientation in the frontal and transverse planes. However, further work needs to be done to determine the exact source of the variation between the systems.

## Limitations

Our results should be interpreted in the context of several limitations. First, of the two systems being compared, neither are considered gold-standard (tantalum beads). Instead, we aimed to compare two methodologies to find convergent validity, as such, there is no way to determine which measurement system is “more accurate”. Instead our results should be interpreted to mean that ankle and MLA angles measured in the sagittal plane between the systems are directly comparable, while out-of-plane angles are more questionable.

The BVR data is rotoscoped manually, leaving it susceptible to intra-tracker errors and resulting in long processing times. Future BVR use would benefit greatly from an automated tracking algorithm, possibly informed by OMC to provide initial pose estimates, and research into the repeatability of this approach. Second, BVR is limited by the available field of view for data collection. The collection space at present is only large enough to examine one joint complex at a time. Our study also only had a relatively small sample size. As we have not used inferential statistics here (which are only appropriate for assessing differences, rather than similarity), performing a sample size calculation is not possible. However, we have provided descriptive statistics which describe similarity. While our sample size is small, an estimate of the standard error of the mean suggests that the margin for the mean RMS angles or marker positions would be approximately 0.52° or 0.37 mm across both conditions.

Finally, the processing times of the two systems is notably different. The BVR approach requires segmentation and subsequent rotoscoping of each individual bone, both heavily time intensive processes. When compared to the relatively quick processing of OMC data, which was performed over 1 week—the benefit of directly measuring bone movement may be outweighed by the lengthy processing times. Current understanding of OMC and BVR and their effect on kinematic outputs would benefit from studies with more participants, across a range of foot anatomical types, and compared to the gold-standard (tantalum beads) to assess validity of measures.

## Conclusion

In conclusion, when looking at group mean for ankle angle and MLA angle, OMC and BVR are in good agreement for sagittal plane motion. However, there is a divergence between the systems in the transverse and frontal planes, with the exact cause for divergence unknown. Notably, BVR can provide novel insight into the 3D motion of bones that were previously difficult or impossible to measure. Future work with BVR would benefit from determining the cause of the divergence between the two systems.

## Data Availability

The datasets generated for this study can be found in the University of Queensland eSpace.

## Author Contributions

LK, NK, SD'A, AC, MR, and GL contributed to the conception, design of the study, as well as the data acquisition. SK, GL, MR, and LK organized the database, while SK performed the manual-tracking of the bone data. SK, GL, MR, and LK were responsible for the data analysis and statistical testing. All authors contributed to the manuscript revision, read, and approved the submitted version.

### Conflict of Interest Statement

One or more of the authors (GL, LK, AC) has received funding from the UQ Collaborative Industry Engagement Fund, collaborating with Asics Oceania. One of the authors (SD'A) has received support and resources, as well as, the use of facilities at the Providence VA Medical Center, Providence, RI, USA. The remaining authors declare that the research was conducted in the absence of any commercial or financial relationships that could be construed as a potential conflict of interest.
