# Supplementary material for: A Direct Comparison of Biplanar Videoradiography and Optical Motion Capture for Foot and Ankle Kinematics
Source: Front Bioeng Biotechnol. 2019 Aug 23;7:199. doi: 10.3389/fbioe.2019.00199 (PMC6716496; doi:10.3389/fbioe.2019.00199)
Supplement: Supplementary file 1 [file Presentation_1.pptx]

## Slide 1
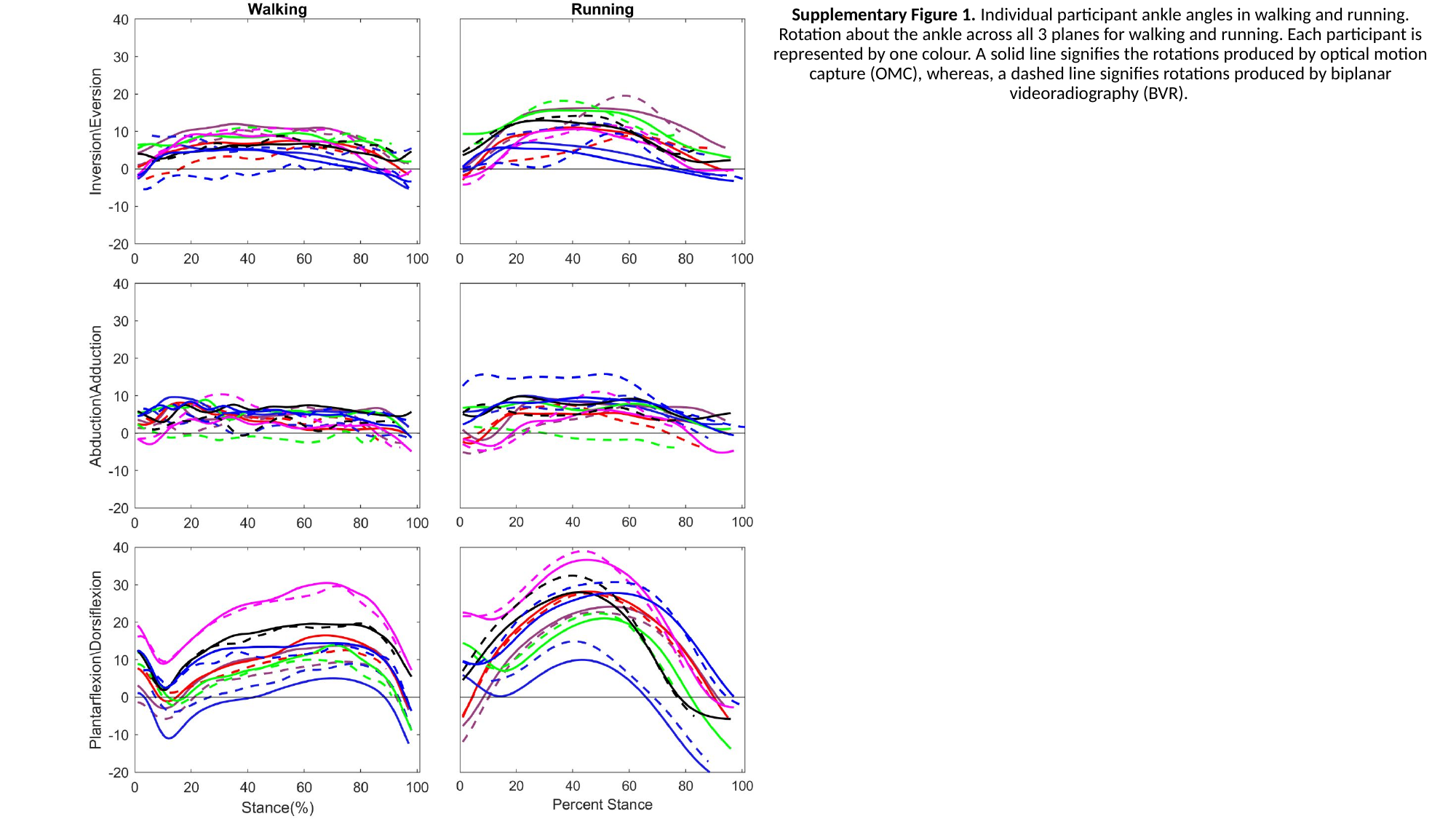

Supplementary Figure 1. Individual participant ankle angles in walking and running. Rotation about the ankle across all 3 planes for walking and running. Each participant is represented by one colour. A solid line signifies the rotations produced by optical motion capture (OMC), whereas, a dashed line signifies rotations produced by biplanar videoradiography (BVR).

## Slide 2
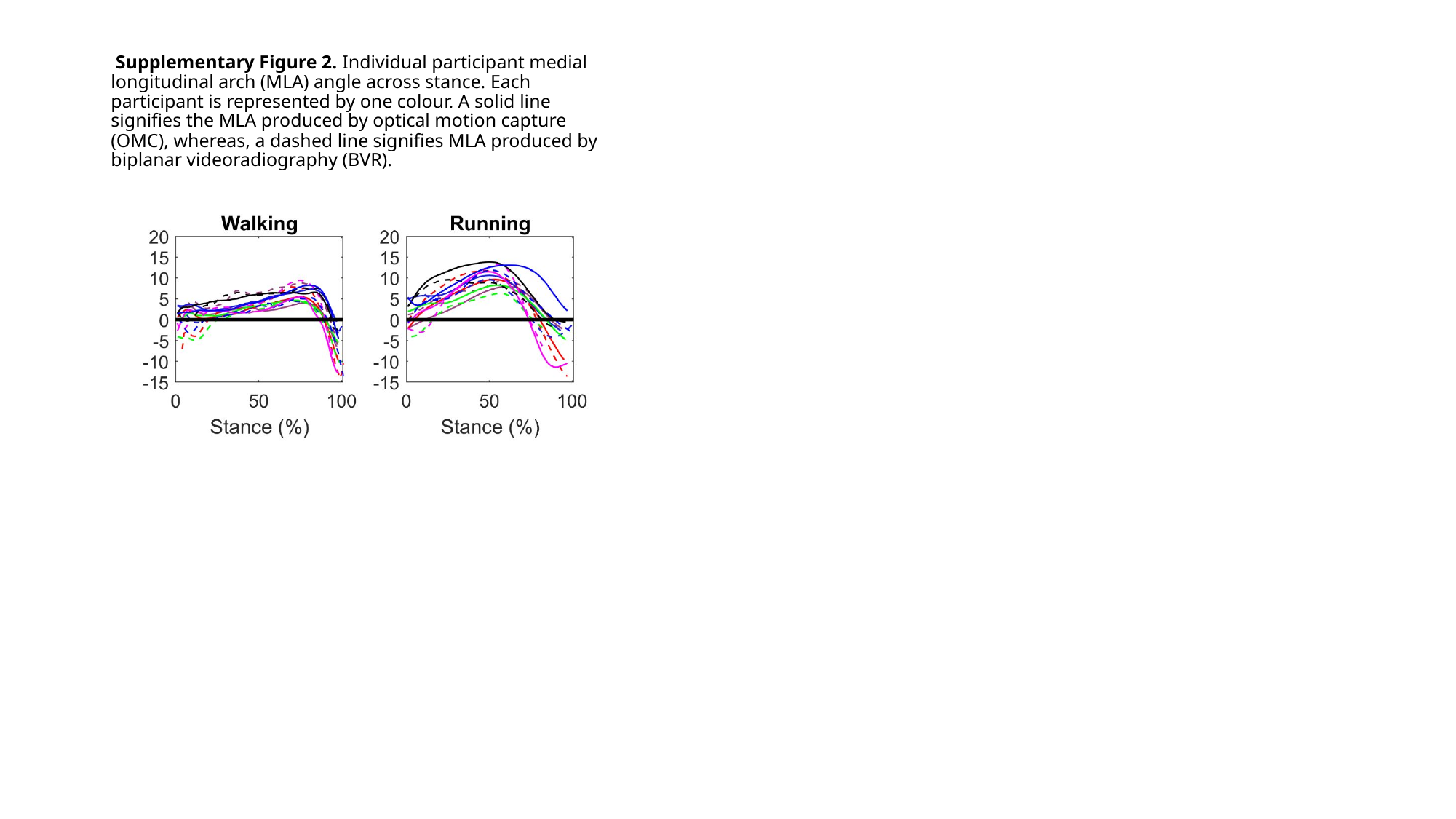

# Supplementary Figure 2. Individual participant medial longitudinal arch (MLA) angle across stance. Each participant is represented by one colour. A solid line signifies the MLA produced by optical motion capture (OMC), whereas, a dashed line signifies MLA produced by biplanar videoradiography (BVR).

## Slide 3
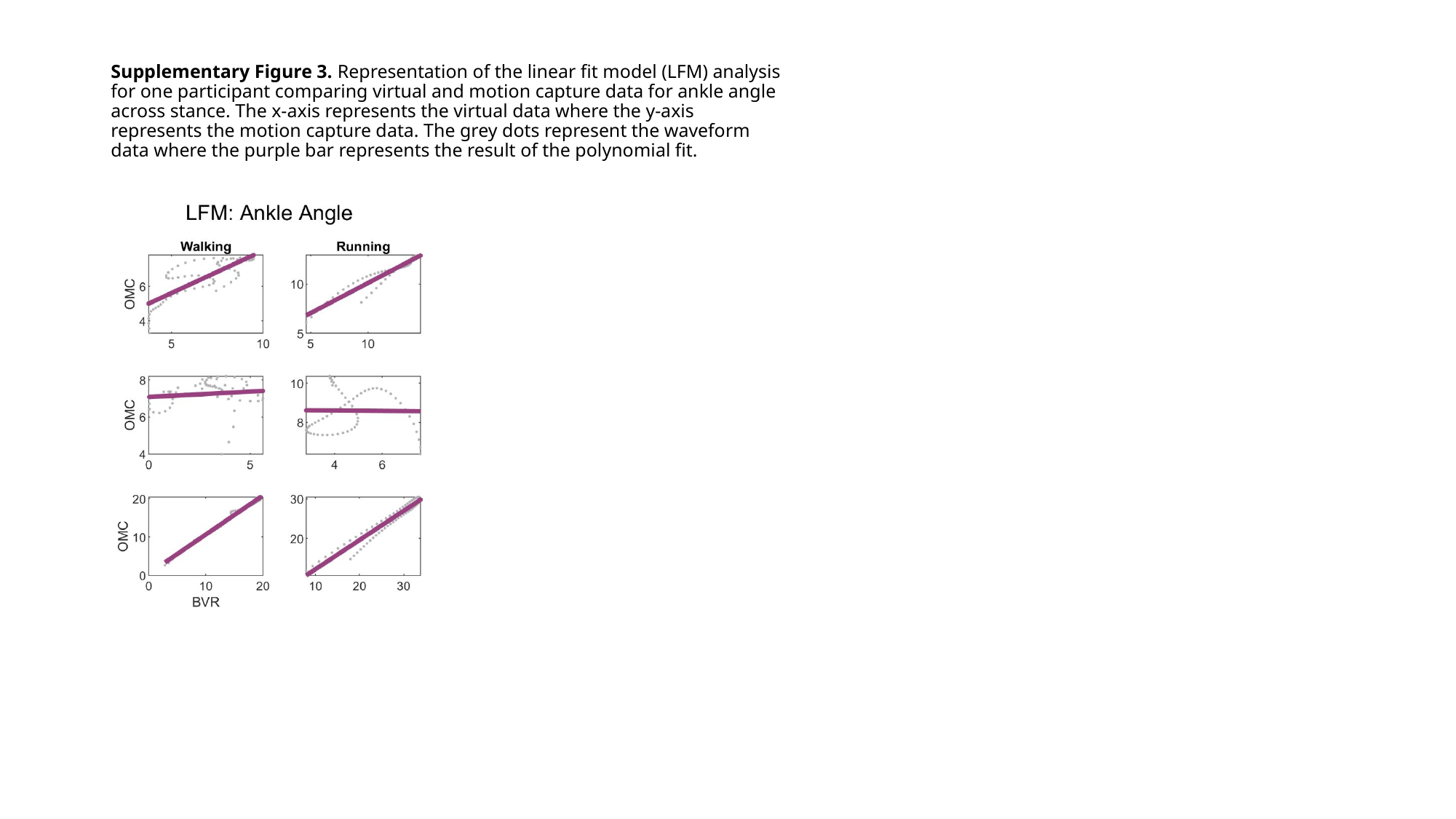

# Supplementary Figure 3. Representation of the linear fit model (LFM) analysis for one participant comparing virtual and motion capture data for ankle angle across stance. The x-axis represents the virtual data where the y-axis represents the motion capture data. The grey dots represent the waveform data where the purple bar represents the result of the polynomial fit.

## Slide 4
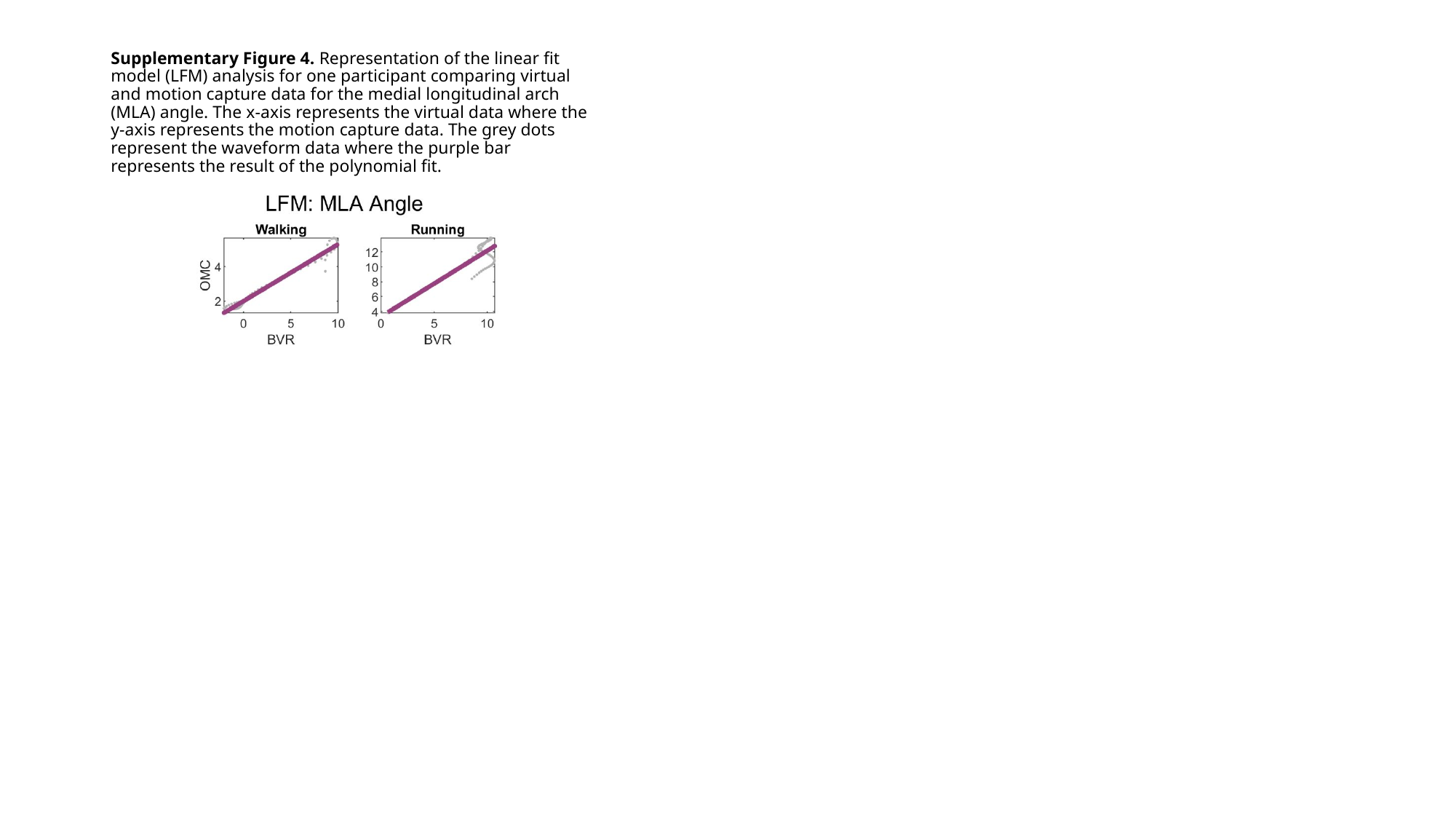

# Supplementary Figure 4. Representation of the linear fit model (LFM) analysis for one participant comparing virtual and motion capture data for the medial longitudinal arch (MLA) angle. The x-axis represents the virtual data where the y-axis represents the motion capture data. The grey dots represent the waveform data where the purple bar represents the result of the polynomial fit.
